# Supplementary material for: A beneficial adaptive role for CHOP in driving cell fate selection during ER stress
Source: EMBO Rep. 2024 Jan 2;25(1):228–53. doi: 10.1038/s44319-023-00026-0 (PMC10897205; doi:10.1038/s44319-023-00026-0)
Supplement: Supplementary file 4 — Source Data Fig. 1 [file 44319_2023_26_MOESM4_ESM.zip › Figure 1 source data/Figure 1A source data/README.rtf]

Nomenclature:example: KO 2 2,2f,5-1_021.fcsGenotype: KnockoutCell line: #2 of 2 for that genotypeStress condition: 2.5 nM TGBiological replicate: #1Sample number: 021example: WT 1 0-2_008.fcsGenotype: Wild-typeCell line: #1 of 2 for that genotypeStress condition: 0 nM TGBiological replicate: #2Sample number: 008Cells labeled “starve” were continued in serum starvation (no ER stress) during the course of the experimentCells labeled “2nd” are secondary antibody-only controls
