# Supplementary figures and images for: A beneficial adaptive role for CHOP in driving cell fate selection during ER stress
Source: EMBO Rep. 2024 Jan 2;25(1):228–53. doi: 10.1038/s44319-023-00026-0 (PMC10897205; doi:10.1038/s44319-023-00026-0)

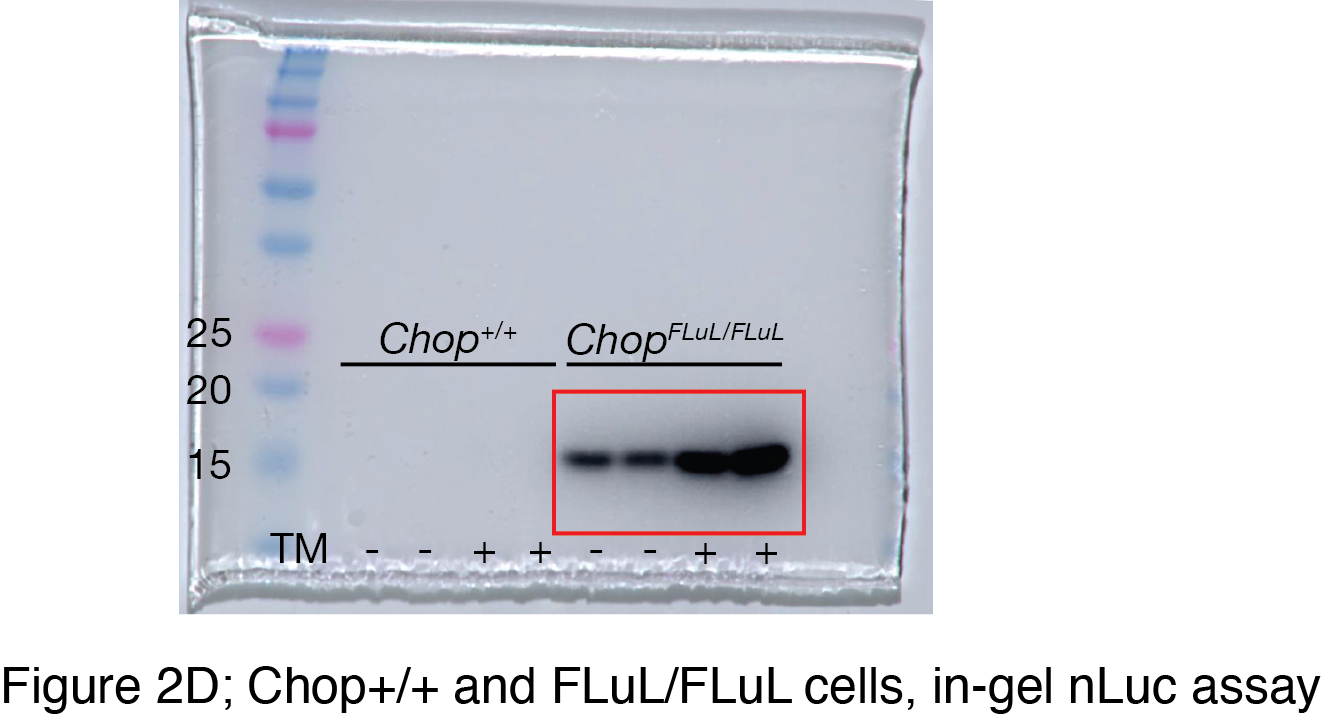

Supplement: Supplementary file 5 — Source Data Fig. 2 [file 44319_2023_26_MOESM5_ESM.zip › Figure 2 source data/Figure 2D source data/Figure 2D source data.png]

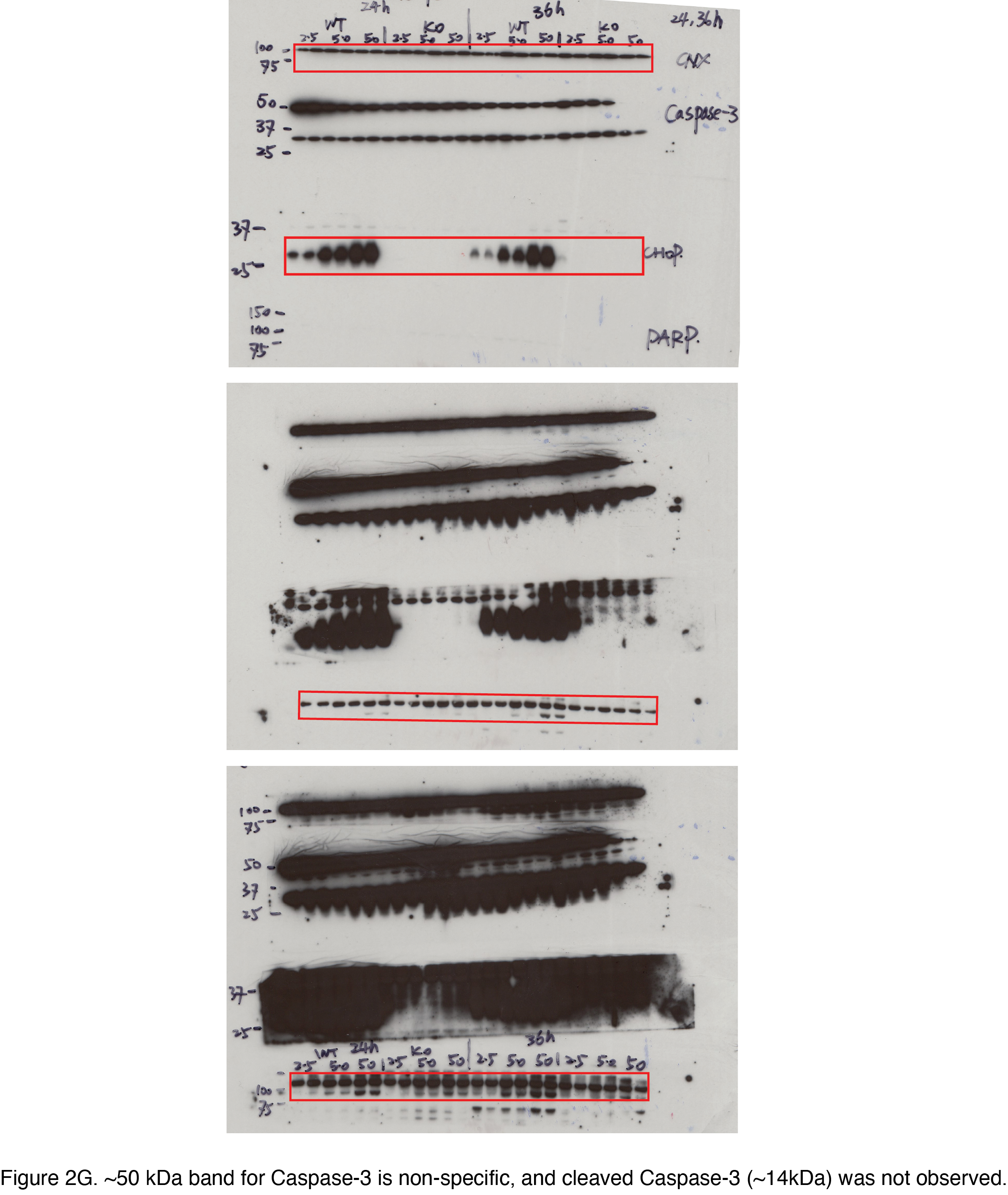

Supplement: Supplementary file 5 — Source Data Fig. 2 [file 44319_2023_26_MOESM5_ESM.zip › Figure 2 source data/Figure 2G source data/Figure 2G source data.png]

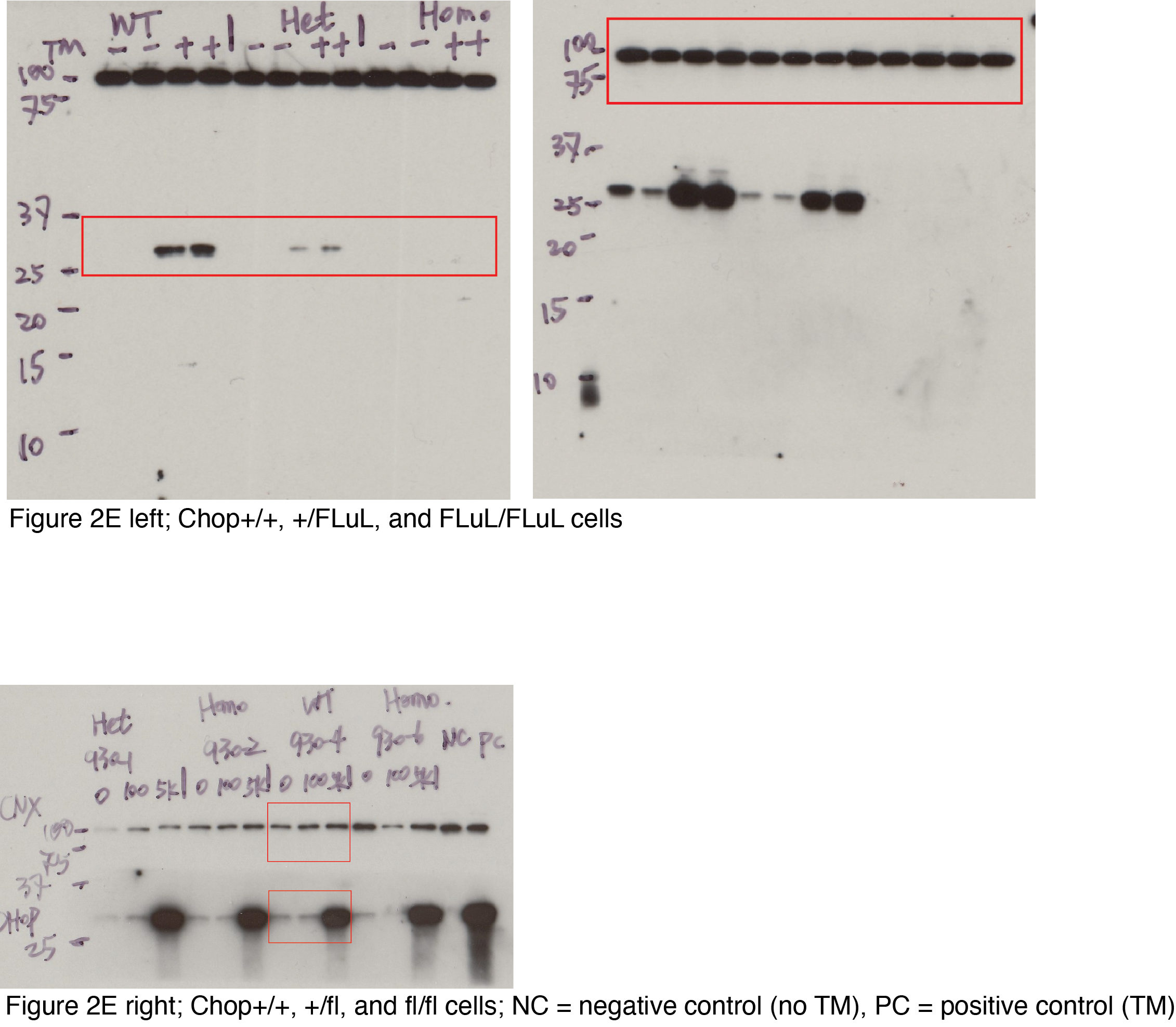

Supplement: Supplementary file 5 — Source Data Fig. 2 [file 44319_2023_26_MOESM5_ESM.zip › Figure 2 source data/Figure 2E source data/Figure 2E source data.png]

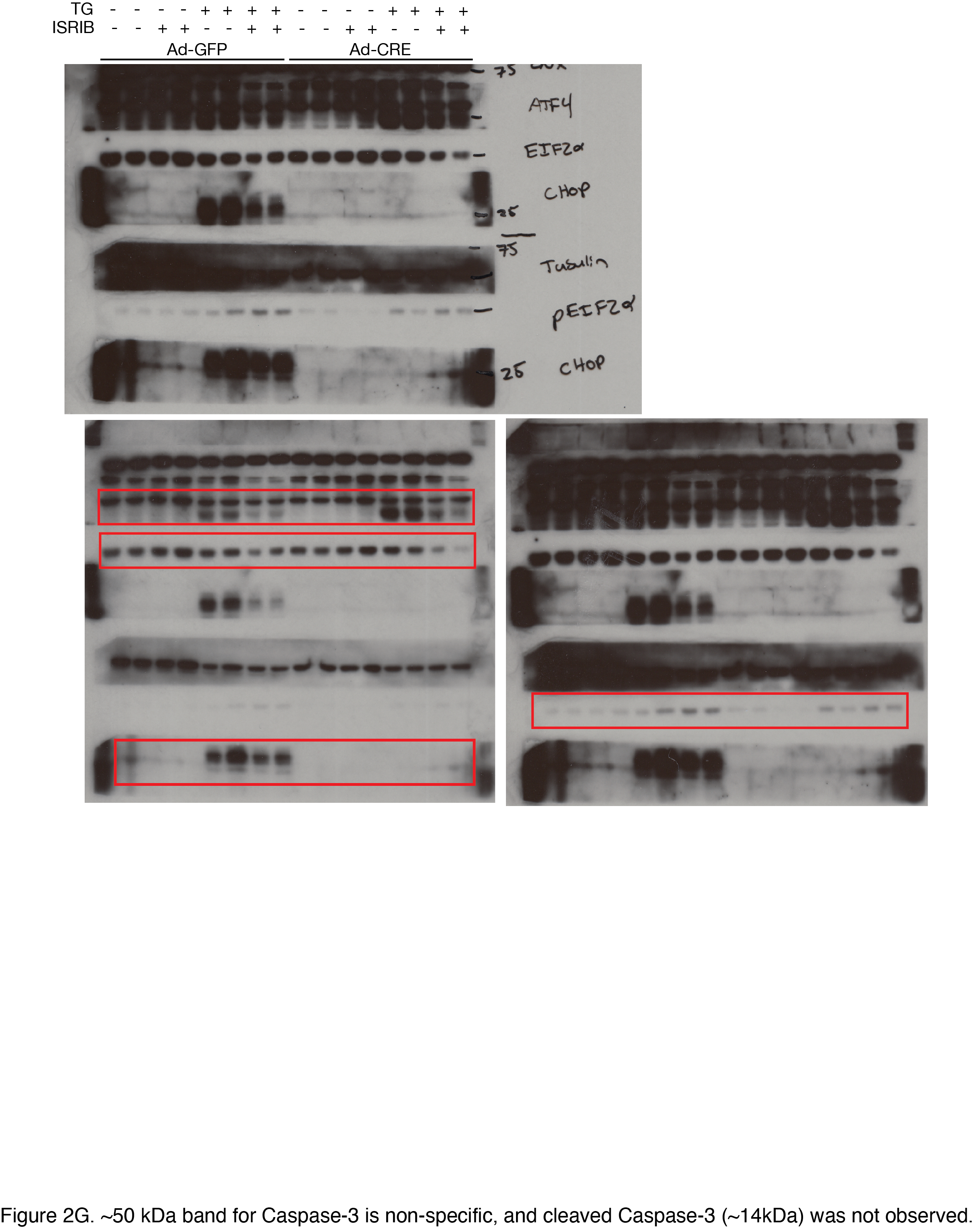

Supplement: Supplementary file 7 — Source Data Fig. 4 [file 44319_2023_26_MOESM7_ESM.zip › Figure 4 source data/Figure 4D source data/Figure 4D source data.png]
