# Supplementary material for: A beneficial adaptive role for CHOP in driving cell fate selection during ER stress
Source: EMBO Rep. 2024 Jan 2;25(1):228–53. doi: 10.1038/s44319-023-00026-0 (PMC10897205; doi:10.1038/s44319-023-00026-0)
Supplement: Supplementary file 6 — Source Data Fig. 3 [file 44319_2023_26_MOESM6_ESM.zip › Figure 3 source data/Figure 3A source data/README.rtf]

Nomenclature:example: KO-2 50 Tg-2_008.fcsGenotype: KnockoutCell line: #2 of 2 for that genotypeStress condition: 50 nM TGBiological replicate: #2Sample number: 008example: WT-1-0_012.fcsGenotype: Wild-typeCell line: #1 of 2 for that genotypeStress condition: 0 nM TGBiological replicate: #1Sample number: 012
