# Supplementary material for: A beneficial adaptive role for CHOP in driving cell fate selection during ER stress
Source: EMBO Rep. 2024 Jan 2;25(1):228–53. doi: 10.1038/s44319-023-00026-0 (PMC10897205; doi:10.1038/s44319-023-00026-0)
Supplement: Supplementary file 11 — Expanded View Figures [file 44319_2023_26_MOESM11_ESM.pdf]

Expanded View Figures

Figure EV1. The effects of CHOP on proliferation and cell cycle regulation in MEFs primary and immortalized MEFs.

(**A**) FACS gating for the experiment shown in Fig. 1A. (**B**) Non-synchronized MEFs were treated with vehicle or 50 nM TG for 24 h and assessed for EdU positivity as in Fig. 1B. Means +/− S.D.M. from independently treated wells. Two-way ANOVA with Šidák, ns = not statistically significant, ****$p < 0.0001$. (**C**) Representative FACS plots of TM data shown in Fig. 1C. (**D**) MEFs were treated with vehicle or 50 nM TG for 24 h, trypsinized, and resuspended in propidium iodide to assess cell cycle stage by DNA content. Means +/− S.D.M. from independently treated wells. Two-way ANOVA with Tukey, **$p < 0.01$; ***$p < 0.001$. (**E**) qRT-PCR was used to assess expression of selected cell cycle-related genes in MEFs treated with vehicle or 50 nM TG for 24 h. Means +/− S.D.M. from independently treated wells. Two-way ANOVA with Šidák. Benjamini-Hochberg correction was applied for multiple hypothesis testing. ns = not statistically significant; *$p < 0.05$; **$p < 0.01$. (**F**) Verification of CHOP knockout in TIB-73 cells (two separate clones each of wild-type and knockout) by immunoblot after treatment with 500 nM TG for 8 h. (**G**) Immunoblot showing induction of CHOP compared to non-treated cells after 16 h of treatment with the indicated concentrations of TG (prior to the onset of PARP cleavage). (**H**) Ad-GFP or Ad-CRE infected *Chop*$^{fl/fl}$ MEFs (see Fig. 3) were immortalized by transfection with large T antigen followed by low density passaging to select for immortal cells. EdU positivity during ER stress was assessed as in Figure EV1B above. Each data point represents results from an independent clone. Error bars are means +/− S.D.M. Two-way ANOVA with Šidák, ns = not statistically significant, ****$p < 0.0001$. (**I**) Three separate clones each of control wild-type or CRISPR-deleted CHOP knockout NIH 3T3 cells were assessed for EdU positivity as with TIB-73 cells in Fig. 1D. Means +/− S.D.M. from independently treated wells. Two-way ANOVA with Šidák, ns = not statistically significant.

 © The Author(s)

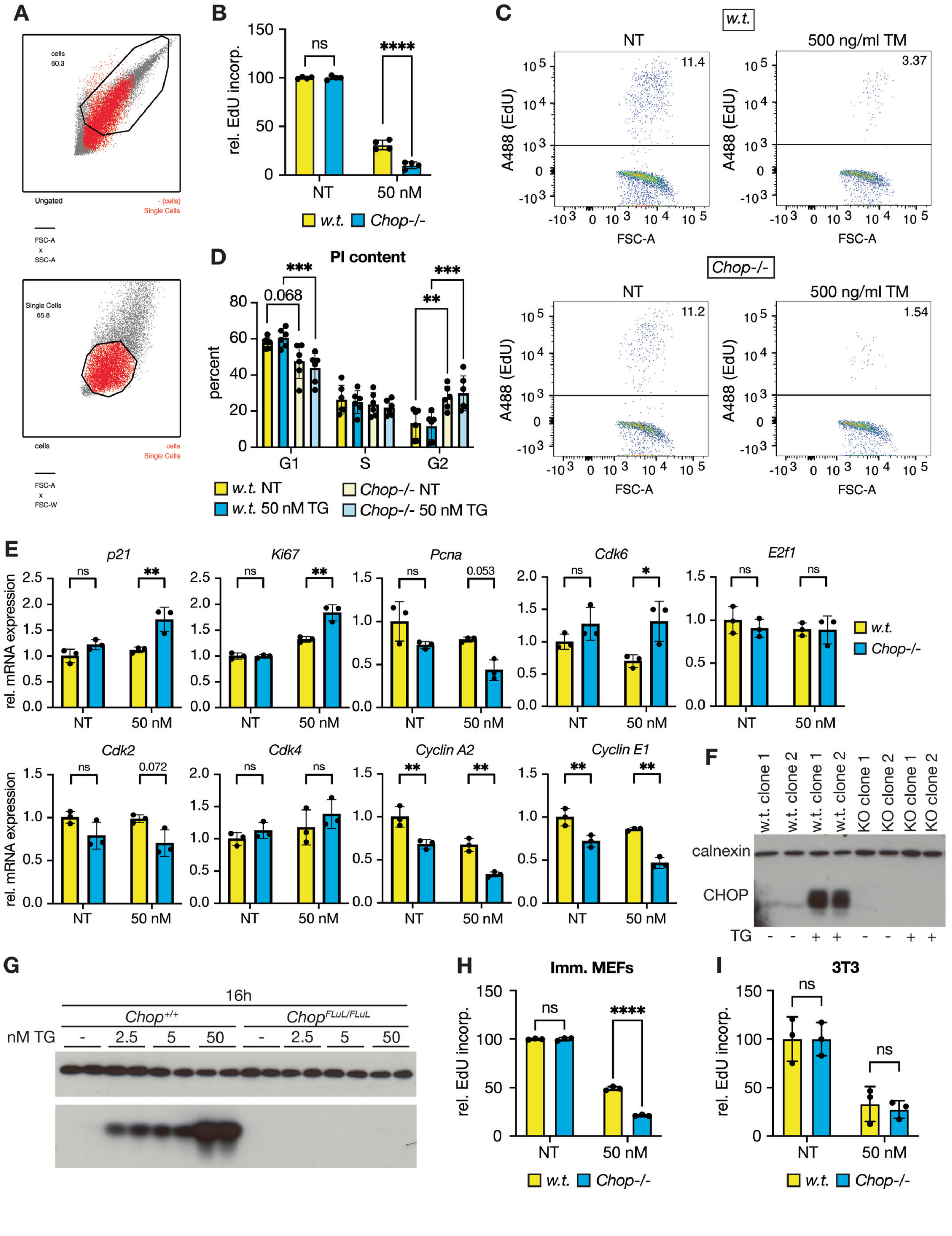
A
cells
60.3
Ungated
- (cells)
Single Cells
FSC-A
x
SSC-A
Single Cells
65.8
cells
cells
Single Cells
FSC-A
x
FSC-W
B
rel. EdU incorp.
ns
****
NT
50 nM
w.t.
Chop-/-
C
w.t.
NT
500 ng/ml TM
11.4
3.37
A488 (EdU)
FSC-A
Chop-/-
11.2
1.54
D
PI content
percent
0.068
***
**
G1
S
G2
w.t. NT
w.t. 50 nM TG
Chop-/- NT
Chop-/- 50 nM TG
E
p21
Ki67
Pcna
Cdk6
E2f1
rel. mRNA expression
0.053
Cdk2
Cdk4
Cyclin A2
Cyclin E1
0.072
F
w.t. clone 1
w.t. clone 2
KO clone 1
KO clone 2
calnexin
CHOP
TG
G
16h
Chop+/+
ChopFLuL/FLuL
nM TG
2.5
5
50
H
Imm. MEFs
I
3T3

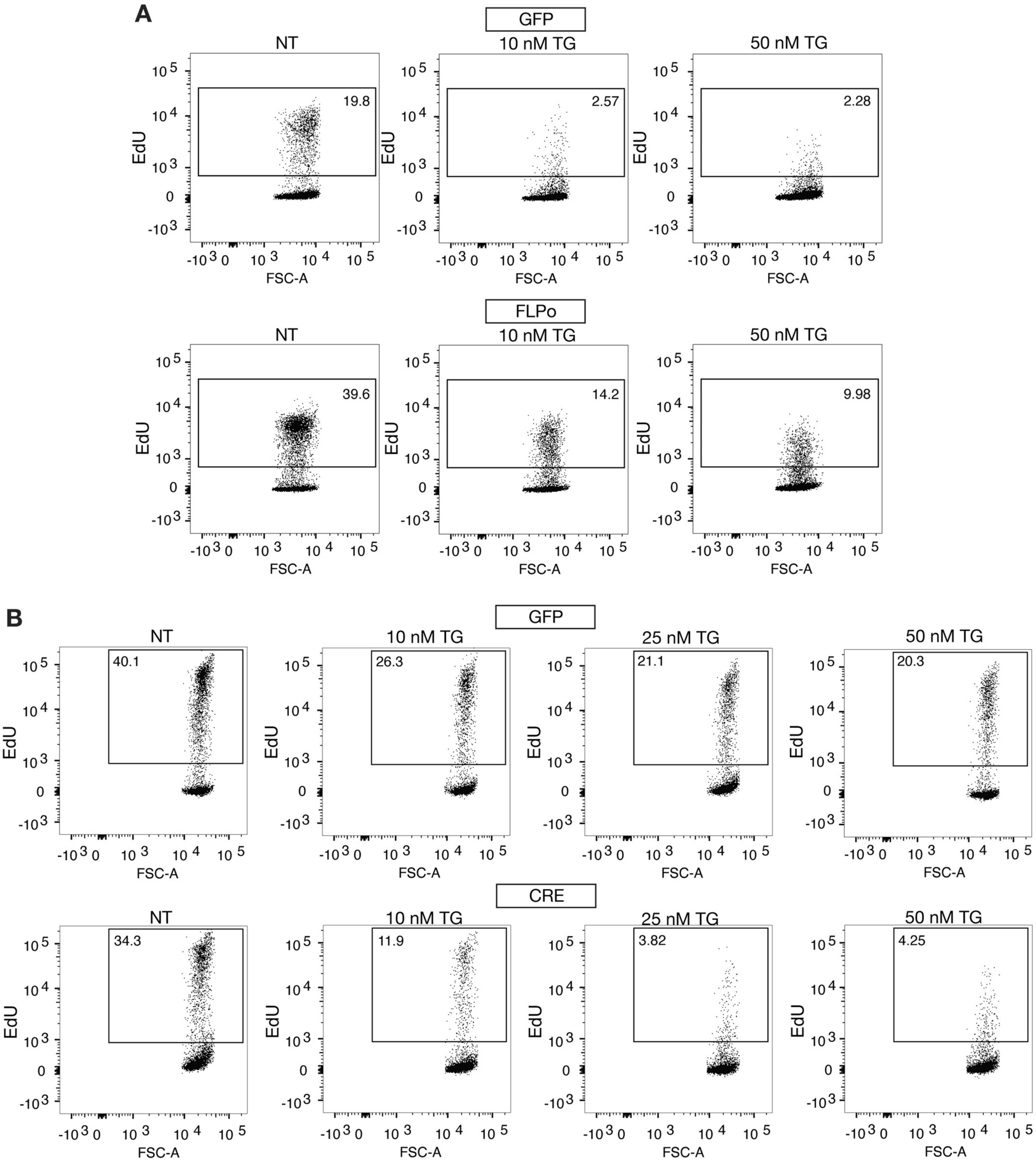

Figure EV2. Individual flow cytometry plots during TG treatment of CHOP-expressing or non-expressing cells.

Representative flow cytometry plots from the bar graphs depicted in Fig. 3C (**A**) and 3D (**B**).

 © The Author(s)

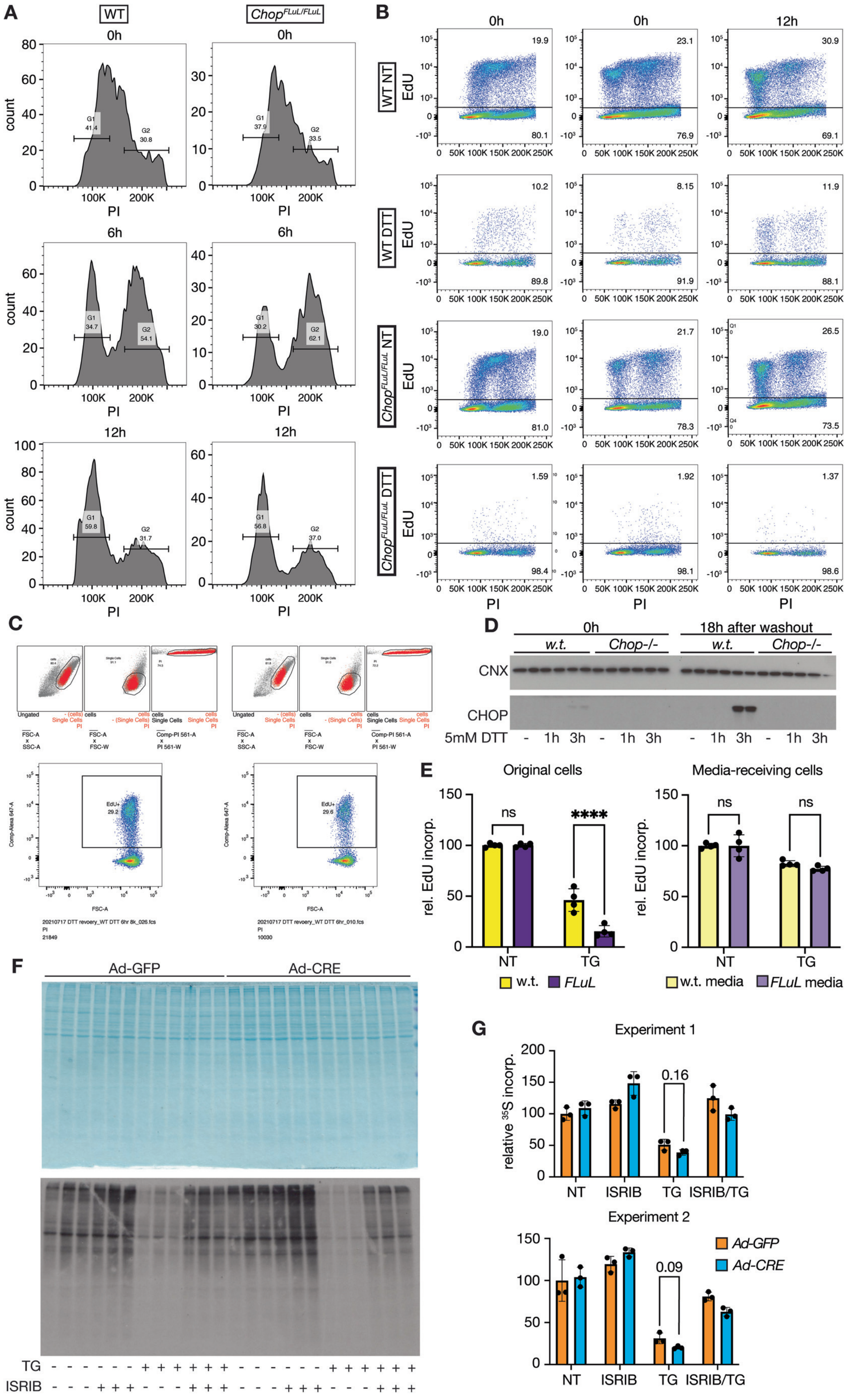
A
WT
Chop$^{FLuL/FLuL}$
0h
6h
12h
count
PI
G1
G2
B
0h
12h
WT NT
WT DTT
Chop$^{FLuL/FLuL}$ NT
Chop$^{FLuL/FLuL}$ DTT
EdU
PI
C
D
0h
18h after washout
w.t.
Chop-/-
CNX
CHOP
5mM DTT
E
Original cells
Media-receiving cells
rel. EdU incorp.
ns
****
NT
TG
w.t.
FLuL
w.t. media
FLuL media
F
Ad-GFP
Ad-CRE
TG
ISRIB
G
Experiment 1
Experiment 2
relative ^{35}S incorp.
0.16
0.09
NT
ISRIB
TG
ISRIB/TG
Ad-GFP
Ad-CRE

Figure EV3. Cell cycle progression, cell autonomy of CHOP, and the effects of ISRIB on protein synthesis.

(**A**) Histograms showing DNA content from all cells (EdU+ and EdU-) from Fig. 4C. (**B**) Independent experiment similar to that conducted in Fig. 4C. (**C**) FACS plots showing gating parameters for cells, single cells, and for PI compensation, respectively (top) and overall measured EdU positivity when different numbers of events were analyzed (>21,000 versus >10,000) (bottom). (**D**) Immunoblot showing CHOP either immediately after treatment with 5 mM DTT for 1 h or 3 h, or 18 h after DTT washout. (**E**) *w.t.* and $Chop^{FLuL/FLuL}$ primary MEFs were treated with vehicle or 50 nM TG for 20 h (original cells). Media were collected from each group and applied to a separate set of non-treated wild-type primary MEFs (media-receiving cells). At the same time, the original cells were then maintained in fresh, stressor-free media. After the media switch, EdU was added directly to both original cells and media-receiving cells, and the cells were harvested 4 h later. Means +/− S.D.M. from independently treated wells. Two-way ANOVA with Šidák, ns = not statistically significant; ****$p < 0.0001$. (**F**) Ad-GFP or Ad-CRE-selected MEFs were treated with 50 nM TG and 1 μM ISRIB as indicated. After 4 h of treatment, ^{35}S-methionine/cysteine was added directly to the media at 200 μCi/ml for 30 min. Lysates were analyzed by Coommassie staining (top) and autoradiography (bottom). (**G**) Quantification of relative labeling efficiency (normalized for total protein load) from two such experiments, conducted independently. Means +/− S.D.M. from independently treated wells. Two-way ANOVA with Šidák with *p*-values shown.

© The Author(s)

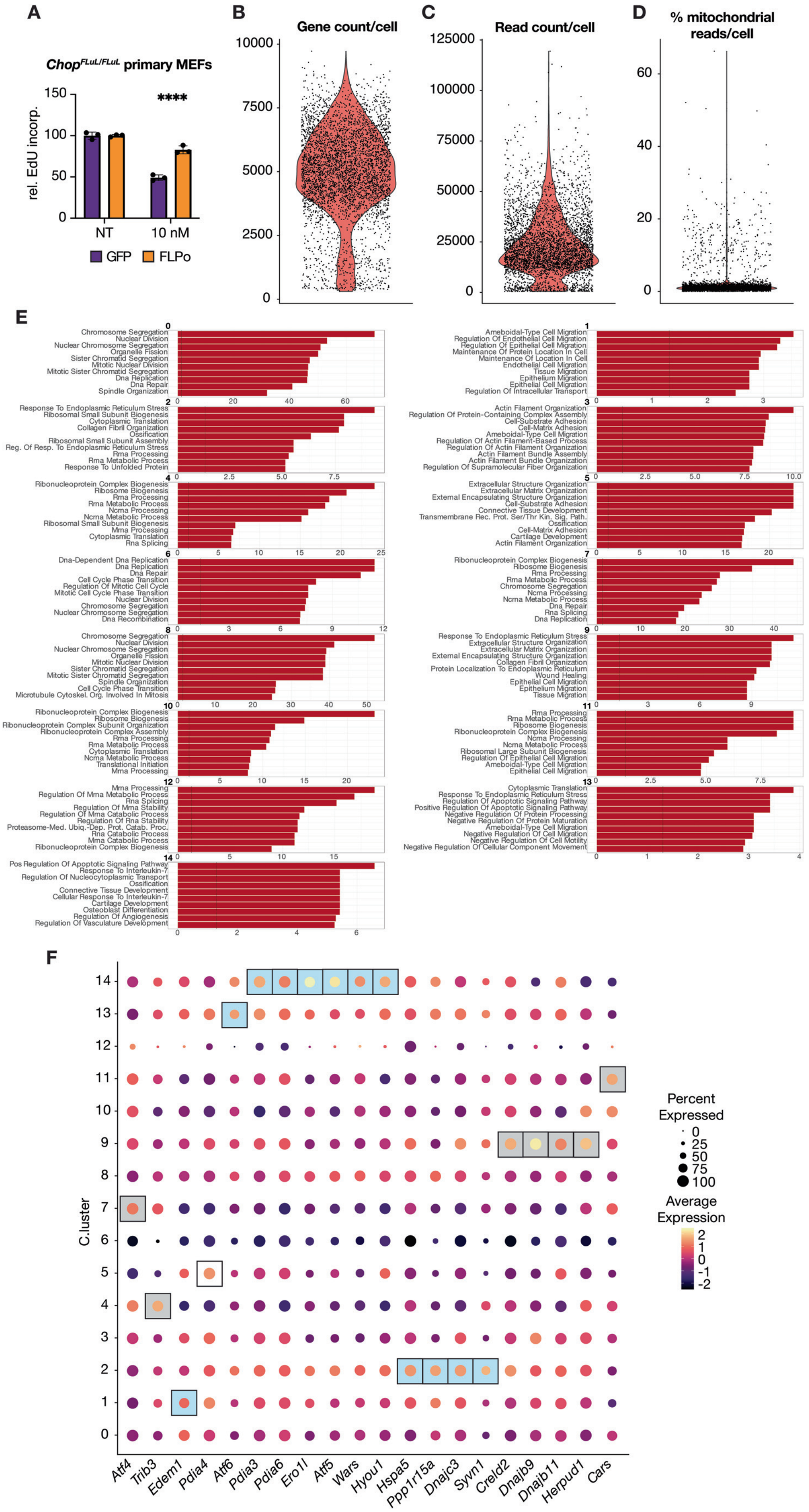
A
Chop^FLuL/FLuL primary MEFs
rel. EdU incorp.
****
NT
10 nM
GFP
FLPo
B
Gene count/cell
C
Read count/cell
D
% mitochondrial reads/cell
E
F
Percent Expressed
Average Expression
Cluster
Atf4
Trib3
Edem1
Pdia4
Atf6
Pdia3
Pdia6
Ero1l
Atf5
Wars
Hyou1
Hspa5
Ppp1r15a
Dnajc3
Syvn1
Creld2
Dnajb9
Dnajb11
Herpud1
Cars

Figure EV4. Extended scRNA-seq validation and pathway analysis.

(**A**) Confirmation of greater EdU positivity in CHOP-expressing (FLPo-treated) cells after treatment with 10 nM TG as in Fig. 7. Means $+/-$ S.D.M. from independently treated wells. Two-way ANOVA with Šidák, ****$p < 0.0001$. (**B–D**) Violin plots showing gene count, read count, and percentage of reads from mitochondrial transcripts, respectively. (**E**) Pathway analysis for each of the 15 clusters from scRNA-seq. The x-axis shows the -$\log_{10}$ *p*-value after correction for false discovery. *P*-values were calculated with enrichGO, by over Representation Analysis and Bonferroni correction for false discovery. (**F**) Relative expression of selected UPR target genes in each cluster is shown. Boxes are used to indicate the cluster in which each gene is most highly expressed, with blue shaded boxes indicating CHOP-positive clusters and gray shaded boxes indicating CHOP-negative clusters (cluster 5 is a mix of both CHOP-positive and -negative cells). Bonferroni correction for false discovery.

 © The Author(s)
